# Analysis of the lateral slope’s impact on the calculation of water-filled rut depth

**DOI:** 10.1371/journal.pone.0243952

**Published:** 2020-12-11

**Authors:** Jiao Yan, Hongwei Zhang, Bing Hui

**Affiliations:** 1 School of Vehicle Engineering, Xi’an Aeronautical University, Xi'an, Shaanxi, China; 2 Key Laboratory of Road Structure and Materials of Inner Mongolia, Hohhot, China; 3 Inner Mongolia Communications Construction Engineering Quality Supervision Bureau, Hohhot, China; 4 School of Highway, Chang’an University, Xi'an, Shaanxi, China; Tongii University, CHINA

## Abstract

Accurate calculation of the water-filled rut depth is critical for assessing hydroplaning potential. Nevertheless, due to the difficulty in collecting and calculating the water-filled rut depth, most transportation agencies do not use i, especially in the case of lateral slopes, although water-filled rut depth is a key parameter that impacts driving safety. Contributions of this paper are development of a methodology to reliably compute the water-filled rut depth and quantitatively evaluate the influence of lateral slope on the water-filled rut depth. The proposed method include: 1) acquisition of the high-resolution 3D point cloud data of rut, 2) smooth processing of rut profile through moving average method with Matlab programming, 3) water-filled rut depth computation at different lateral slopes with the assistance of key points based on rut sections. With the variation of water-filled rut depth (ΔWD), its change rate (δWD), and the calculation error between the rut depth and the water-filled rut depth (Δ_n_) as evaluation indexes, the variation law of water-filled rut depth under different lateral slopes is analyzed when considering the severity level and rut shape of the rut profile. Results show that: 1) the increase in lateral slope leads to the reduction of water-filled rut depth; 2) the water-filled rut depth is affected by the rut shape, including rut side wall’s slope and the key points’ elevation. The accurate calculation of the water-filled rut depth can provide reliable suggestions for safe driving.

## 1. Introduction

The accumulation of water in the rut road section poses a threat to the safety of vehicles. The water in the rut will cause part or even all of the friction between the tire and the road surface to lose, resulting in safety hazards such as vehicle hydroplaning and sliding. For this reason, it is necessary to measure the water-filled rut depth accurately to ensure the reliability of vehicle safety analysis. The rut depth measured by the wire method or the straightedge method [1,2] is the maximum water depth, that is, the depth of the rut in the dry state, and the influence of the lateral slope (cross slope and super elevation) on the water-filled rut depth is ignored. The water-filled rut depth is not only determined by the single factor of rut shape. The water-filled rut depth of the same shape varies under different lateral slopes, which leads to the difference in the rut depth and safety risk. When the rut depth exceeds 7.6 mm, the rut-related accident rate begins to increase at a significantly greater rate [3]. In addition, every 2.5 mm increase in the rut depth raises the car crashes by 16%, as reported by Cenek [4] in the United States. Thus, the accurate measurement of rutting depth’s impact on traffic accidents analysis should not to be ignored, not to mention the water-filled rutting depth. Therefore, the influencing factors of water-filled rut depth need to be further investigated.

As an important indicator of road design, lateral slope (cross slope and super elevation) provides turning centripetal force and affects road cross-section drainage, but it is seldom mentioned in the standards for rut evaluation and maintenance. Due to the limitations of current detection technology, it is hard to detect complete transverse pavement profiles and lateral slopes on a large scale in the road network level. Most of the previous studies used low intensity, low accuracy Transverse Profilograph or gauges to collect complete transverse pavement profiles and cross-slopes [5]. In addition, there isn’t a unified method for calculating the water-filled rut depth. For example, the rut width and the cross-slope angle were used to calculate the water-filled rut depth by sinusoidal calculation [6,7]. This calculation method simplifies the rut profile and is not suitable for various actual rut profiles. Furthermore, the elevation of the section point was adopted to obtain the distance between the water edge and the lowest water point, so as to calculate the water-filled rut depth [5,8–10]. This method is reasonable theoretically, but only the concept of this approach is proposed, and few studies have analyzed its practical application. Due to the difficulty of cross-slope and rut profile detection, the water-filled rut depth is rarely used to evaluate rut severity or analyze driving safety.

In addition, although the importance water-filled rut depth is increasingly recognized, the difference between the water-filled rut depth and the dry rut depth has not been quantitatively analyzed. Luo [11] calculated the hydroplaning speed and identified the potential hydroplaning road segments by using the water-filled rut depth. From the analysis results, it is known that the potential hydroplaning road segments divided by the water-filled rutting depth and non-water-filled rutting depth is not the same, but there is no quantitative analysis. Simpson [12] reported that the volume of accumulated water is a function of the rut depth, the cross slope, and the longitudinal slope. This dependency is well understood, however, no method is provided to calculate the water-filled rut depth.

The above research shows that due to the limitation of detection techniques and the inconsistent definition, water-filled rut depth has not been applied to the road network by transportation departments, despite being considered to be important for driving safety. Furthermore, the influence of lateral slope on water-filled rut depth is still not quantified. Therefore, by advanced 3D line laser technology, full-profile rut point cloud elevation can be collected [13], and the accurate measurement of water-filled rut depth can be obtained. The purpose of this paper is to explore the influence of lateral slope on water-filled rut depth, and to demonstrate the necessity of lateral slope detection in rut maintenance.

## 2. Rut morphology and data collection

### 2.1 Rut shape and its deterioration

Pavement rutting, also referred to as permanent deformation, is a “contiguous longitudinal depression deviating from a surface plane defined by transverse cross slope and longitudinal profile” [1,14,15]. It is a “permanent or unrecoverable traffic-associated deformation within pavement layers” [16]. It can arise because of the densification effect from repetitive traffic loading; it can also be caused by design or structural failure of the surface and/or supporting layers of the pavement, or by construction quality issues.

Deterioration of pavement rut has been extensively studied, mainly for the purpose of pavement design. Existing models predict the growth of rut depth over time and cumulative traffic loading, either empirically by correlating field observations (e.g., traffic configuration, weather conditions, and other surface distresses) with the growth of rut, or mechanistically by deriving theoretical pavement layer responses via structural analysis means and laboratory tests [16–18].

**Fig 1:**
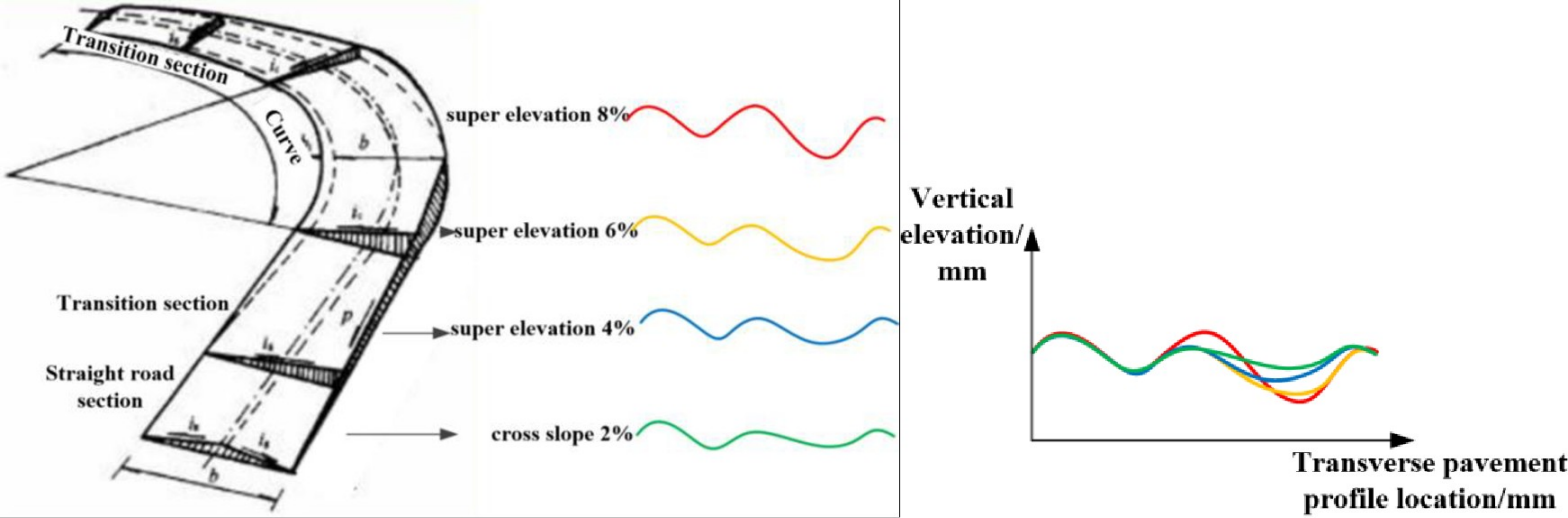
Ruts on straight and curved road sections.

The impact of lateral slopes (including cross slope and super elevation) on rut deterioration is analyzed below. As shown in Fig 1, the cross slope is usuallly designed and constructed to be 1–2%, the super elevation on curves are lightly lager, but no bigger than 8% in the expressway [19]. For straight road sections, the right rut depth is genrally larger than the left (right rudder). The wheel track on the outer side of the lane bear a greater load due to the cross slope. When a car is running on left-turn curved road sections, the wheel track on the outer side of the lane bears a greater load; at the same time the centripetal force becomes larger. Rut profiles at different super elevations on curves are different. When lateral slope increases, the required centripetal force increases, so does the rut depth on the right side.

### 2.2 Data acquisition and pre-processing

**Fig 2:**
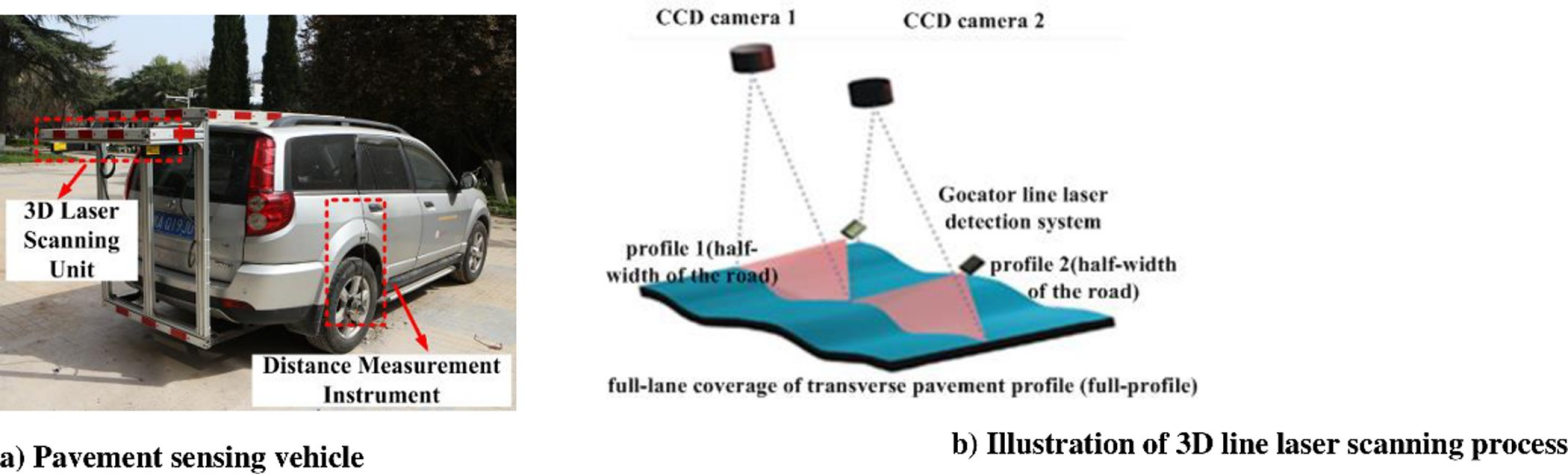
3D laser sensing vehicle of Chang’an University and full-lane detection of rut.

As shown in Fig 2A), the 3D pavement surface data are collected by the 3D laser scanning vehicular system (3D LSVS). The 3D LSVS is equipped with two laser scanning units and a high-resolution Distance Measurement Instrument (DMI). Among them, the DMI which is mounted on the right rear wheel controls the data collection interval between two consecutive transverse profiles. Two laser scanning units (Gocator 2380) are used to continuously collect 3D transverse pavement profiles. These two laser units are approximately 1.8 m above the ground. They can acquire profile data from two separated sensors with 1,280 data points covering a 3.5 m wide lane. The transverse pavement profile consists of two sets of profile data 1 and 2, which constitutes the profile data of the whole lane (Fig 2B).

The 3D LSVS needs to avoid rainy days given that the testing results will be affected by water and mud, etc. When the driving speed is below 40 km/h, the laser scanning unit, controlled by the DMI, can collect data at 2 mm interval between two 3D transverse profiles in the driving direction at a scan frequency of 4,500 profiles per second. In addition, the 3D laser scanning units provide a 2 mm resolution in the transverse direction (X direction) and 2 mm resolution in the longitudinal direction (Y direction). Laboratory and field tests demonstrated that the 3D LSVS meets the technical requirements specified in PP70-10 [20] and produces data for computing rut parameters.

During the detection, the lateral slope, tire pressure, pavement surface conditions, vehicle trajectories, resolution of the data collection triggering device, and other operational and systematic variation, differences in 3D pavement data collected at different timestamps are unavoidable [21]. Thus, 3D pavement data need to be registered by a semi-automated method according to Wang’s research. The vertical elevation of the inner side of left and right lane marking is zero after the registration.

In order to ensure the accurate calculation of water-filled rut depth, smoothing pretreatment of removing outliers and noise is required for the detected transverse pavement profile. According to AASHTO PP69 [2], the moving average filter is used to smooth the elevation point cloud data of rut profile detected by 3D laser (Eq 1).

$${\text{z'}}_{\left[i\right]}=\frac{1}{m}\sum_{j=-(m-1)/2}^{\left(m-1\right)/2}z_{\left[i+j\right]}$$ (1)

**Fig 3:**
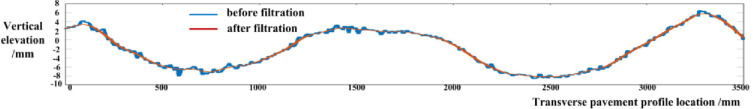
Rut profile before and after filtration.

Where z_[]_ is the input data, $z_{\left[\right]}^{'}$ is the filtered output data and m is the size of filtered window with the length of 50 mm (when calculating the rut depth) and 200 mm (when calculating the water-filled rut depth) [2]. As shown in Fig 3, rut profiles before and after filtration are illustrated by blue and red lines, respectively.

### 3. Methodology of rut parameters calculation

**Fig 4:**
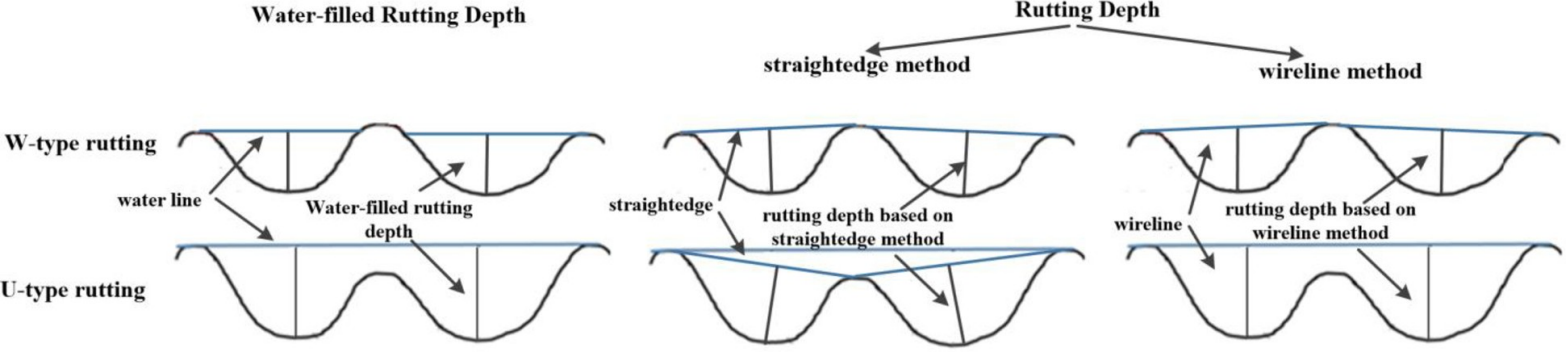
Rut parameters calculation methods.

Fig 4 illustrates the rut parameters calculation methods for different types of rut. Here, the W-shaped rut refers to rut with a crown in the middle, and the U-shaped rut refers to rut without a crown. According to the definition of AASHTO PP69 [2], the water-filled rut depth is determined by the elevation difference between the water spillover point and the lowest elevation point of section, as is manifested in the vertical distance between the lowest elevation point and water line. Rut depth can be calculated by the straightedge and wireline methods, especially the straightedge method, two major approaches to derive rut depth from collected transverse profiles [22]. According to the *Standard Test Method for Measuring Rut-Depth of Pavement Surfaces Using a Straightedge*, the distance between the bottoms surface of the straightedge and the pavement after the gauge is measured as the rut depth. Moreover, according to the wireline method stipulated in Figure T 0973–3 in JTG E60-2008 *Field Test Methods of Subgrade and Pavement for Highway Engineering* [23], the maximum vertical distance between the wire and the rut profile on both sides is calculated, that is, the rut depth. In this paper, the wireline method is adopted since its definition is more close to the water-filled rut depth, especially when U-shaped rut is considered.

**Fig 5:**
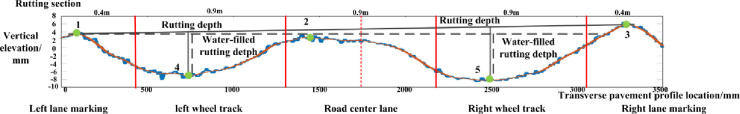
Schematic diagram for the calculation of rut parameters and rut section.

Fig 5 is an example for the calculation of rut depth and water-filled rut depth. As is mentioned above, the key points with green circle below are identified in the calculation of rut depth and the water-filled rut depth with the help of auxiliary line (including wire line and water line). The key points include maximum and minimum points. The former determines the contact point of the wireline with the rut profile and the edge point with water overflow, while the latter determines the lowest point of wireline method and the lowest point of accumulated water. Owing to the fluctuation of rut profile, there may be one or more extreme points. It is therefore necessary to adopt the secondary screening to obtain the maximum value according to the road partition where the extreme value points are located.

To determine the key points of the rut profile, the road surface is divided into five regions by defining the wheel track. This paper employs the definition of wheel-track given by Florida Department of Transportation [24] to divide lanes, as shown in Fig 5. The whole lane is divided into five areas by the two wheel-tracks with the length of 0.9 m. The distance from the wheel track to the lane marking is 0.4 m, and the distance between the two wheel tracks is 0.9 m.

The key points of rut are extracted according to the divided rut sections, and the maximum points are screened twice through the maximum language in Matlab (Eqs 2 and 3). These maximum points are located outside the wheel track zone, while the minimum points are in the section of the wheel track. The maximum points are labelled 1, 2 and 3 from left to right, and the minimum points are labelled 4 and 5.

transverselocationofthemaximumpointsindtop=find(diff(sign(diff(z)))==-2)+1; (2)

transverselocationoftheminimumpointsindbottom=find(diff(sign(diff(z)))==2)+1; (3)

Where “z” is the vertical elevation of the laser points on the rut profile.

As shown in Fig 5, the connecting line between point 1 and point 3 is wire line, and the line extending from point 1 to its right is water line. The left and right rut depths are equal to the vertical distance from point 4 and point 5 to the wire line respectively. The left and right water-filled rut depths correspond to the vertical distance from point 4 and point 5 to the water line.

## 4. Case study

The objective of this paper is to analyze the impact of the single variable lateral slope on water-filled rut depth. Thus, rut profiles with similar shapes but different lateral slopes on straight road and curved road respectively need to be collected and compared. This assumption is reasonable because there is such a possibility in large number of rut profiles from the prospective of road network-level. Neverthelsss, rut on curved road cannot be collected so far.

Due to the limitation of experimental conditions, this paper adopts the rotation method to obtain the rut profiles with lateral slopes for hypothesis analysis, and attempts to analyze the difference between the rut depth and the water-filled rut depth at different lateral slopes. This paper aims at doing feasibility study that trying to arouse the attention of scholars and engineers in analyzing the safety driving states of water-filled rut with lateral slopes.

### 4.1 Data preparation

**Fig 6:**
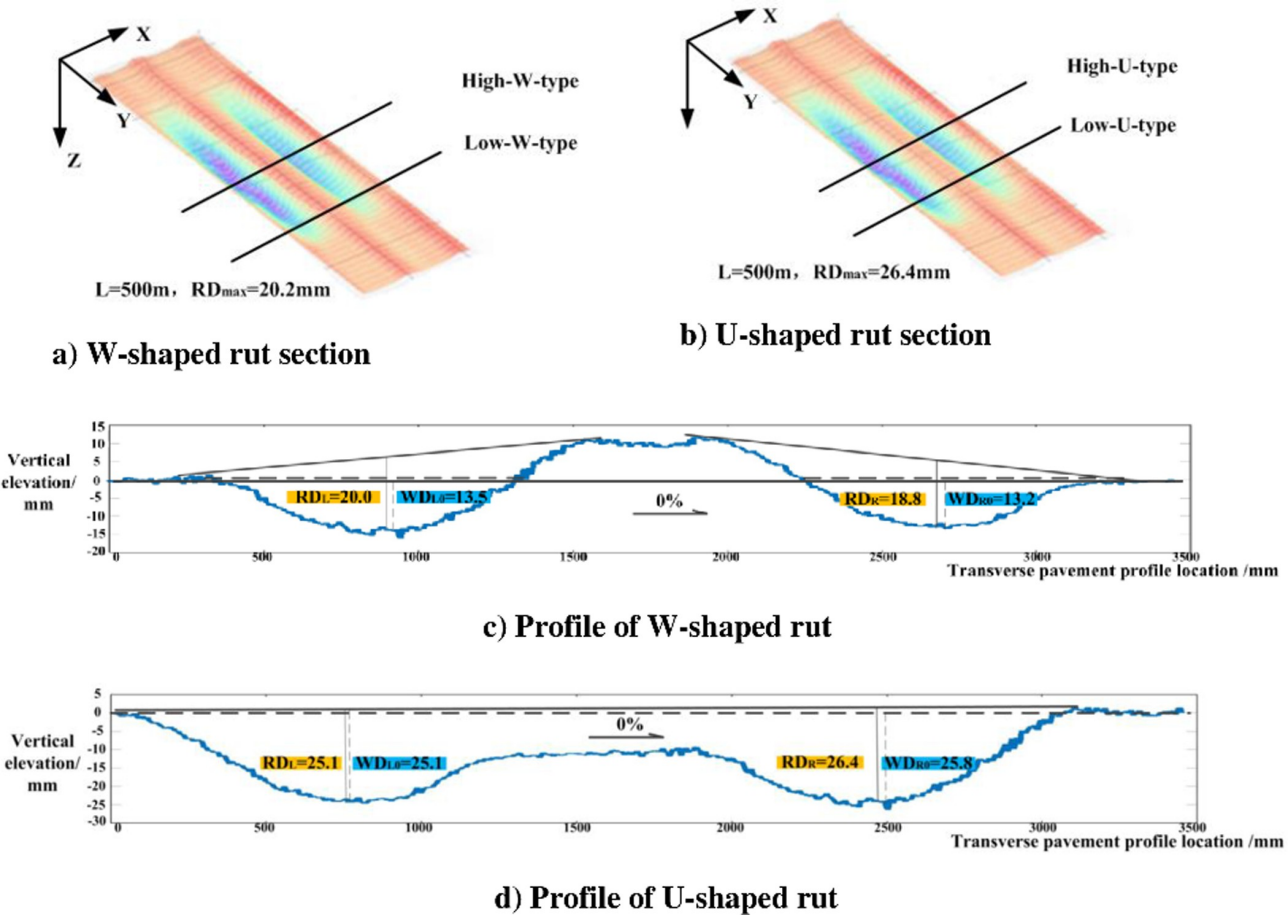
Characteristic profile of ruts.

The elevation information of continuous adjacent profiles with different rut shapes in the two straight road sections of 500 meters long in provincial highway can be obtained by Chang’an University sensing vehicle. Moreover, four profiles of low and high severity levels are selected for analysis, among which 10–15 mm refers to low-severity rut and 15–25 mm high-severity rut according to the *Highway Performance Assessment Standards* [25] of China. Rut A (in Fig 6A and 6C) is a W-shaped rut with the maximum depth of 20.2 mm and Rut B (in Fig 6B and 6D) is a U-shaped rut with the maximum depth of 26.4 mm, which need to be repaired by maintenance department according to the *Technical Specifications for Maintenance of Highway Asphalt Pavement* [26] of China.

In an attempt to analyze the influence of lateral slope on the water-filled rut depth, the rut profile are rotated by 2%, 4%, 6% and 8% respectively (represented by *α*) according to Eqs 4 and 5. Then the automatic calculation of the water-filled rut depth at different lateral slopes is realized with the help of Matlab.

$$x_{k}^{'}=x_{1}+(x_{k}-x_{1})\cos\alpha +y_{k}\sin\alpha$$ (4)

$$y_{k}^{'}=(x_{k}-x_{1})\sin\alpha -y_{k}\cos\alpha$$ (5)

The vertical elevation difference △H between left and right ends after the rotation of rut profile is presented in Table 1. RW shows the horizontal distance between left and right endpoints of the rut profile.

ΔH=RW*tan(α) (6)

**Table 1:** Influence of lateral slope on the elevation difference between ending points.

*α*	2%	4%	6%	8%
△H(mm)	70	140	210	280

### 4.2 Selected evaluation methods

After comparing the current evaluation indexes [27–31], in the present paper, the impact of lateral slope on the depth calculation is evaluated by the variation of water-filled rut depth (ΔWD) and the variation rate of water-filled rut depth (δWD). △WD can quantitatively respond to the change in accumulated water and has a high correlation with safety. δWD can intuitively describe the change in accumulated water without considering the influence of rut severity levels. The equations are expressed as follows.

$${\Delta \text{WD}}_{\text{n}}{=\text{WD}}_{\text{n}}{-\;\text{WD}}_{\text{0}}$$ (7)

$${\delta \text{WD}}_{\text{n}}=\left({\text{WD}}_{\text{n}}{-\;\text{WD}}_{\text{0}}\right){/\text{WD}}_{\text{0}}$$ (8)

Where WD_0_ is the water-filled rut depth of the registered rut profile with a lateral slope of 0%, and WD_n_ denotes the water-filled rut depth with specified lateral slopes. WD_0_ is the maximum water-filled rut depth, which is compared with the ruts of other slopes.

In addition, the deviation between the rut depth (RD) and the water-filled rut depth (WD) is analyzed, denoted by Δ_n_ (Eq 9).

$$\Delta_{\text{n}}{=\text{RD}}_{}{-\;\text{WD}}_{\text{n}}$$ (9)

### 4.3 Results analysis

Table 2 compares the ruts of low and high severity levels, and shows the results of the variation of water-filled rut depth (ΔWD) and its change rate (δWD) at different lateral slopes.

**Table 2:** Variation of the water-filled rut depth and its change rate.

No.	Severity level	0%		Lateral slope 2%		Lateral slope 4%		Lateral slope 6%	
		WD_L0_ (mm)	WD_R0_ (mm)	△WD_L2_ (mm)	△WD_R2_ (mm)	△WD_L4_ (mm)	△WD_R4_ (mm)	△WD_L6_ (mm)	△WD_R6_ (mm)
		WD_L0_ (mm)	WD_R0_ (mm)	δWD_L2_	δWD_R2_	δWD_L4_	δWD_R4_	δWD_L6_	δWD_R6_
1#	Low-U-shape	14.4	12.4	10.4	9.6	13.2	11.3	14.4	12.4
				0.73	0.78	0.92	0.91	1	1
2#	High-W-shape	13.5	13.2	1	8.8	8.5	10.7	11.6	13.2
				0.07	0.67	0.63	0.81	0.86	1
3#	High-U-shape	25.1	25.8	19.5	10.8	22.9	20.4	25.1	22
				0.78	0.42	0.91	0.79	1	0.85
4#	Low-W-shape	5.5	5.2	-6.1	3.3	1.2	5.2	5.5	5.2
				-1.11	0.64	0.22	1	1	1

First, whether the water-filled rut depth will be affected by the change in lateral slope is studied by sensitivity analysis. Variance analysis is the most commonly used approach for sensitivity analysis [32]. Herein, the one-way variance analysis is employed with lateral slope as control variable, and variation of accumulated water depth Δ WD as observation variable. The significance level is 0.05, and the variance analysis results are given in Table 3.

**Table 3:** Impact of lateral slope on variation of water-filled rut depth.

			Quadratic sum	df	Mean square	F	Significance
Differences between groups	(Combination)		7.913	12	0.659	6.819	0.000
	Sums of square	Comparison	7.620	1	7.620	78.795	0.000
		Deviation	0.293	11	0.027	0.276	0.987
Differences within groups			3.771	39	0.097		
Total			11.684	51			

It can be seen from Table 3 that, when only the lateral slope is considered, the obtained F is 6.819 and the corresponding probability *p* = 0<0.05 (significance level). This indicates that there is a significant difference in the variation of accumulated water depth under different lateral slopes. It is thus can be concluded that lateral slope has a great impact on the accumulated water depth.

The water-filled rut depth varies in a range of (-6.1, 25.1) mm. Besides, the difference between the rut depth and the water-filled rut depth ranges within (0, 26.4) mm. Table 2 also shows that water-filled rut depth varies considerably regardless of rut severity levels. The wading test indicates that vehicle hydroplaning will occur when the water film is 3 mm in thickness [14]; the study conducted by Start shows that the water-filled rut depth of 7.6 mm and the length of 9.1 m contribute to the occurrence of vehicle hydroplaning; according to Cenek [4], every 2.5 mm increase in rut depth will raise accident rate by 16%; Norwegian research shows that every 5–10 mm increase in water-filled rut depth will increase the probability by 5% [4]. This demonstrates that the accurate measurement of water-filled rut depth’s impact on traffic accidents analysis should not to be ignored.

**Fig 7:**
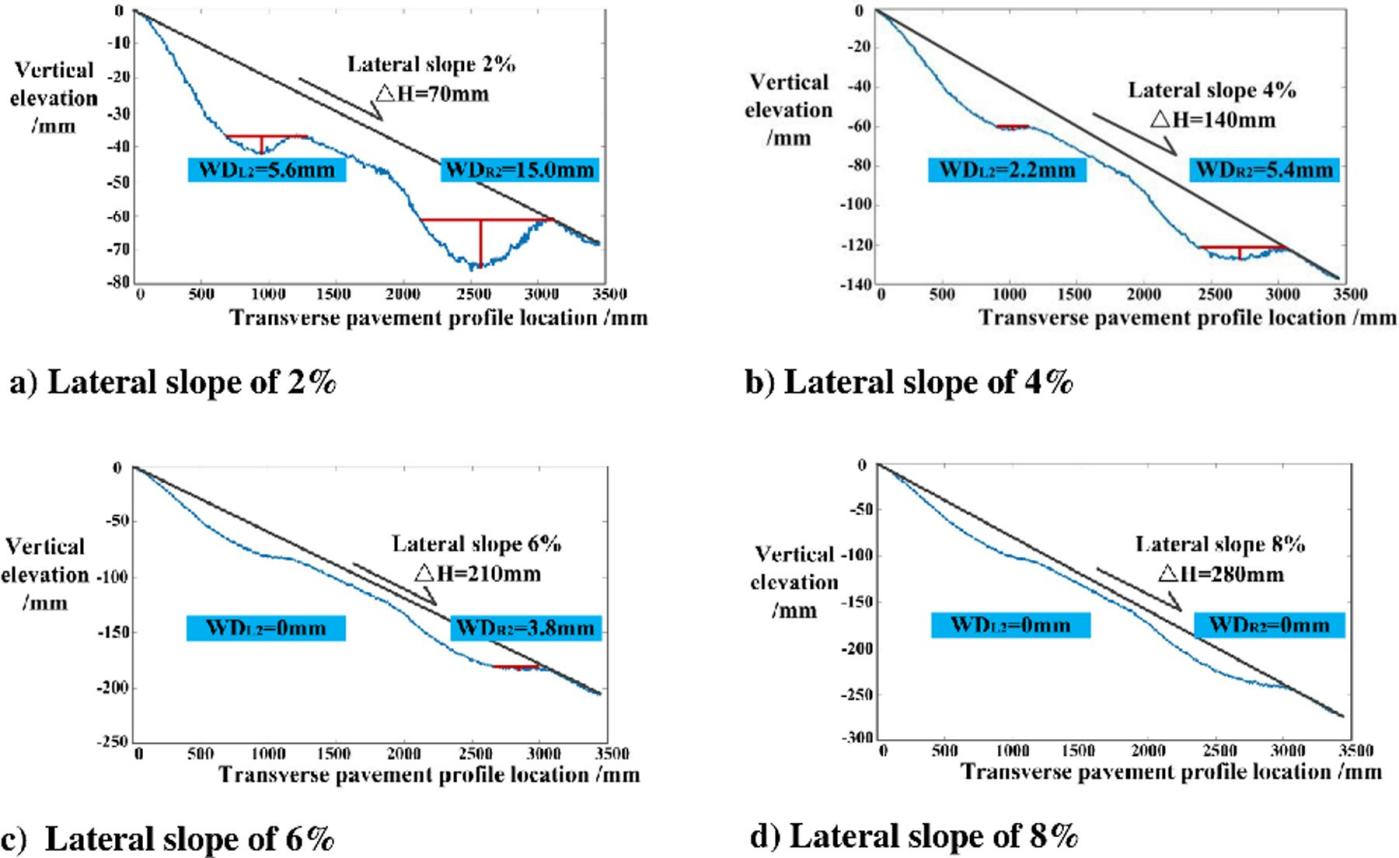
Variation of water-filled rut depth with lateral slope.

Taking U-shaped Rut 3 # as an example, Fig 7 presents the variation of water-filled rut depth with the lateral slope of 2%, 4%, 6% and 8% respectively. It can be found that the accumulated water gradually decreases with the rising lateral slope. With regard to the lateral slope of 6%, the amount of accumulated water of left ruts is 0 mm, while in the case of lateral slope of 8%, the water amount of both left and right ruts is reduced to 0 mm. Based on the calculation above, the water-filled rut depth of all ruts is 0 with the slope of 8% and thus is not presented in Tables 2 and 4. From the variation of accumulated water volume, it can be known that lateral slope has a large impact on water depth, which lowers the accuracy of results and finally leads to the mis-grading of rut severity level.

**Table 4:** Comparison of rut depth and water-filled rut depth. (unit: mm).

No.	Severity level	Rut depth		0%		Lateral slope 2%		Lateral slope 4%		Lateral slope 6%	
		RD_L_	RD_R_	△_L0_	△_R0_	△_L2_	△_R2_	△_L4_	△_R4_	△_L6_	△_R6_
1#	Low	14.6	12.8	0.18	0.36	10.62	9.99	13.41	11.61	13.58	12.78
2#	High	20.0	18.8	6.5	5.6	7.5	14.4	15	16.3	18.1	18.8
3#	High	25.1	26.4	0	0.6	19.5	11.4	22.9	21	25.1	22.6
4#	Low	14.3	12.3	8.8	7.1	2.7	10.4	10	12.3	14.3	12.3

The change rate of water-filled rut depth has little correlation with the rut severity level. For example, the severity level of rut is 2 # and 4 # respectively with the change rate of 0.85 and 0.77 in terms of low and severe right rut. The rate is indeed closely correlated with the rut shape. It is obvious that the change rate of W-shaped rut is smaller than that of U-shaped rut, owing to the large Z value at key point 3, even after rotation. This further indicates that the ruts without a ridge in the middle are greatly affected by lateral slope. Actually, among all kinds or ruts, such ruts without ridges account for the largest proportion, leading to the mis-grading of over 50% of rut severity levels.

**Fig 8:**
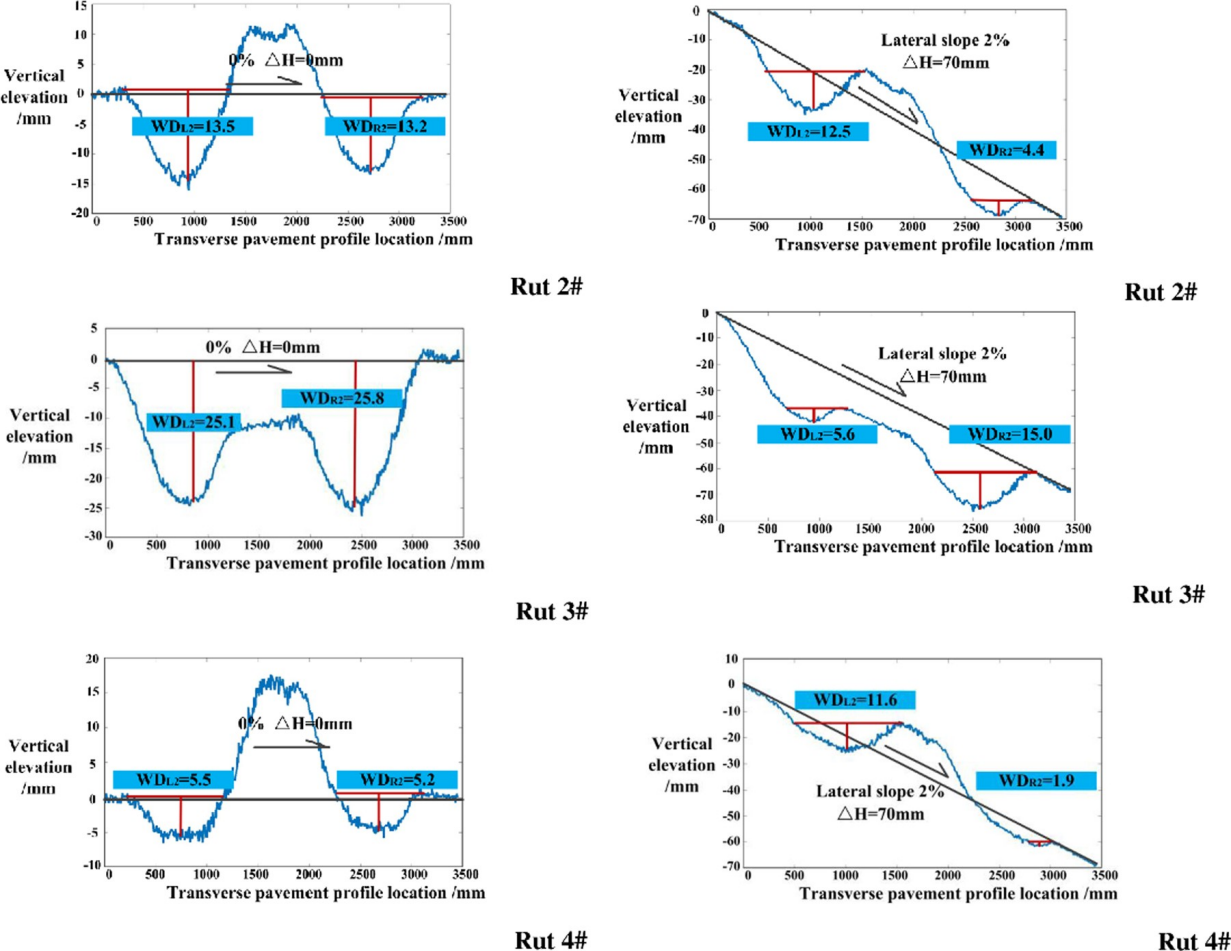
Change in the water-filled rut depth (taking 0% to 2% change of lateral slope as an example).

Specifically speaking, the change mentioned above is influenced by the key points of 1, 2, 3, 4 and 5. With the lateral slope of 0% and 2% as an example (as shown in Fig 8), the water-filled rut depth of the left rut is affected by the points 1, 2 and 4. With regard to the right rut, the water-filled rut depth is influenced by points 3 and 5. Moreover, water is easily discharged after the rotation of lateral slope with a gentle slope of the side walls. Therefore, whether water can be accumulated in the rut is determined by the lateral slope of right rut side wall, as well as the elevation values of points 2 and 3. The larger the cross slope, the greater the vertical elevations of points 2 and 3 and the stronger the water accumulation capacity.

Based on the assessment, rut shape characteristics, including the position, slope, depth, and patterns of a rut will impact the water-filled rutting depth calculation accuracy. It should be noted that the above analyses are all about right-turn curve, so the left side end of the rut profile is higher than the right side end. In addition, drivers on the curve are right rudder. When these rut profiles are on the left-turn curve, the water-filled rut depth will be influenced by the left rut side wall and the elevation values of points 1and 2. Additional actual transverse profiles with different rut shapes on different curves should be further analyzed with regression analysis method, using to quantify the potential errors and further understand the impact of rut shape on water-filled rutting depth error.

## 5. Conclusions and recommendations

In an attempt to evaluate the influence of the lateral slope (cross slope and super elevation) on the water-filled rut depth, the present paper employs 3D laser to obtain the high-resolution point cloud data and calculates the water-filled rut depth under different lateral slopes. With the variation of water-filled rut depth (ΔWD), the change rate of water-filled rut depth (δWD), and the calculation error (Δ_n_) of water-filled rut depth as evaluation indicators, a thorough analysis is conducted on the variation law of the three indices under different lateral-slope conditions and severity levels.

1. The increase in lateral slope leads to the decrease in water-filled rut depth. The variation of water-filled rut depth (ΔWD) and calculation error (Δ_n_) range can be (-6.1, 26.4) mm, which will cause misjudgment in hydroplaning potential evaluation.
2. The water-filled rut depth is influenced by rut profile shape, including rut side wall and the key points’ elevation, which depends on the vertical elevation of key points 2 and 3 and the slope of the right rut wall.
3. The rut depth cannot be simply adopted to express the water-filled rut depth, which is of great significance to pavement rut maintenance decision.

Further research may be carried out on the interaction mechanism between lateral slope and rut deterioration based on practical data. In addition, the influencing factors of water-filled rut depth, such as pavement texture, rainfall intensity, and longitudinal grade need to be further explored. Also, vehicle driving influencing factors like speed needs to be considered in further research.

## Supporting information

S1 File (XLSX)

S2 File (XLSX)
